# Structural characterization of recombinant IAV polymerase reveals a stable complex between viral PA-PB1 heterodimer and host RanBP5

**DOI:** 10.1038/srep24727

**Published:** 2016-04-20

**Authors:** Christopher Swale, Alexandre Monod, Laura Tengo, Alice Labaronne, Frédéric Garzoni, Jean-Marie Bourhis, Stephen Cusack, Guy Schoehn, Imre Berger, Rob WH Ruigrok, Thibaut Crépin

**Affiliations:** 1 Université Grenoble Alpes, Unit of Virus Host Cell Interactions, UMI 3265 UJF-EMBL-CNRS, 71 avenue des Martyrs, CS 90181, F-38042 Grenoble Cedex 9, France; 2 CNRS, Unit of Virus Host Cell Interactions, UMI 3265 UJF-EMBL-CNRS, 71 avenue des Martyrs, CS 90181, F-38042 Grenoble Cedex 9, France; 3 Institut de Biologie Structurale (IBS), Univ. Grenoble Alpes, CEA, CNRS, 38044 Grenoble, France; 4 EMBL Grenoble, 71 avenue des Martyrs, CS 90181, F-38042 Grenoble Cedex 9, France; 5 The School of Biochemistry, University of Bristol, Clifton BS8 1TD, United Kingdom

## Abstract

The genome of influenza A virus (IAV) comprises eight RNA segments (vRNA) which are transcribed and replicated by the heterotrimeric IAV RNA-dependent RNA-polymerase (RdRp). RdRp consists of three subunits (PA, PB1 and PB2) and binds both the highly conserved 3′- and 5′-ends of the vRNA segment. The IAV RdRp is an important antiviral target, but its structural mechanism has remained largely elusive to date. By applying a polyprotein strategy, we produced RdRp complexes and define a minimal human IAV RdRp core complex. We show that PA-PB1 forms a stable heterodimeric submodule that can strongly interact with 5′-vRNA. In contrast, 3′-vRNA recognition critically depends on the PB2 N-terminal domain. Moreover, we demonstrate that PA-PB1 forms a stable and stoichiometric complex with host nuclear import factor RanBP5 that can be modelled using SAXS and we show that the PA-PB1-RanPB5 complex is no longer capable of 5′-vRNA binding. Our results provide further evidence for a step-wise assembly of IAV structural components, regulated by nuclear transport mechanisms and host factor binding.

The influenza virus is a negative sense RNA virus with a segmented genome belonging to the *Orthomyxoviridae* family. The viral RNA (vRNA) is divided into 8 segments that encode for a total of 10 core proteins and a few additional accessory proteins. Every vRNA segment is coated by numerous nucleoprotein (NP) units and by one RNA-dependent RNA-polymerase (RdRp) complex composed of PA (716 aa for influenza A virus (IAV) strains), PB1 (756 aa for IAV strains) and PB2 (757aa for IAV strains). The RdRp binds to the 5′- and 3′- terminal extremities of the vRNA which acts as a promoter region^1^,^2^,^3^. The macromolecular assembly between the vRNA, NP and the RdRp forms the ribonucleoprotein (RNP). The RdRp performs both replication and transcription of the vRNA genome in the nucleus of the cell. As such, the RdRp can produce either cRNA/vRNA through *de novo* replication or viral mRNA through “cap snatching” transcription^4^. The factors regulating the switch from a transcriptive to a replicative RdRp remain to be characterised.

Recently, the structures of influenza B^5^, bat influenza A^6^ and influenza C^7^ were published, providing tremendous insight into the complex architecture of the RdRp. All three subunits are tightly intertwined and form intricate quaternary structures at the vRNA promoter binding sites. Current models of RdRp assembly suggest that PB2 is imported via the importin-α pathway^8^,^9^ whereas the PA-PB1 heterodimer is imported through direct interaction with RanBP5^10^,^11^,^12^.

Structural studies of RdRp from the human infecting influenza A (human-IAV) strains in contrast, have been limited to date, partly due to difficulties to produce recombinant H3N2 or H5N1 polymerases. Nonetheless, a cryo-EM reconstruction was recently obtained of a truncated influenza A/H5N1 polymerase^13^. In the present article, we identify PB2 as the main bottleneck hampering complete recombinant polymerase expression in our insect cells expression system. Moreover, we present the characterisation of several constructs of human-IAV RdRp, including the biologically relevant PA-PB1 heterodimer in solution. The titration measurements against the 5′- and 3′-vRNA promoters show a strong sub-nanomolar affinity of the PA-PB1 heterodimer towards the 5′-vRNA whereas the specific binding of the 3′-vRNA requires the presence of PB2. By applying our co-expression strategy, we show that the previously proposed PA-PB1-RanBP5 import complex can be purified, which we characterise using small angle X-ray scattering (SAXS). Biochemical analysis of this complex reveals a role for RanBP5 in hindering 5′-vRNA binding. Taken together, these data provide evidence for a cellular RdRp assembly pathway following a sequential and conditional process of assembly.

## Results

### PB2 is a key limiting factor for recombinant expression of the heterotrimer

**Figure 1:**
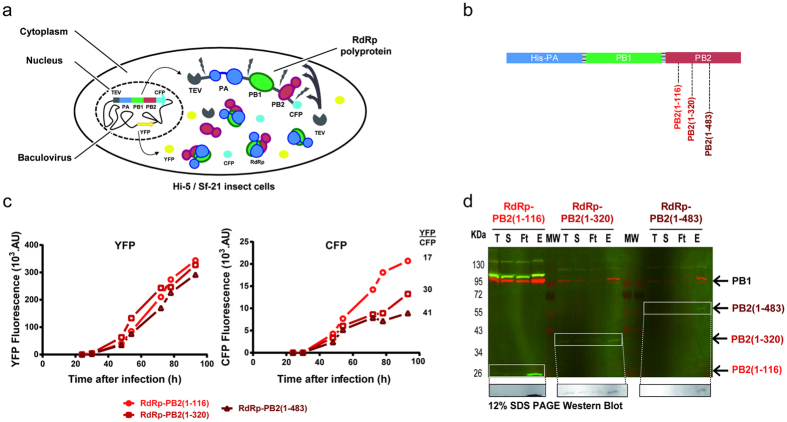
Truncated human-IAV RdRp polyprotein expression. (**a**) Logic of the TEV-PA-PB1-PB2-CFP polyprotein expression in a schematic view. During expression, TEV will process the polyprotein ensuring a stoichiometric assembly of PA, PB1 and PB2. YFP and CFP are produced during the process and monitor respectively baculovirus proliferation and polyprotein translation. (**b**) Truncated RdRp constructs where PB2 is incrementally extended until residues 116, 320 or 483. (**c**) YFP (left) and CFP (right) fluorescence kinetics measured during Hi-5 insect cells culture of truncated RdRp constructs. YFP (λ_exi_ = 488 nm, λ_emi_ = 525 nm) and CFP (λ_exi_ = 430 nm, λ_emi_ = 480 nm) measurements were performed on cellular extracts prepared by sonicating 1 × 10^6^ cells in PBS (500 μL) follow by centrifugation. Fluorescence intensities are plotted against time after infection. (**d**) Small scale nickel resin purification analysis by western blot. Purifications were performed on the 50 mL of Hi-5 insect cells cultures used for the YFP and CFP fluorescence kinetics (**c**). Deposits feature total lysate after freeze/thaw (T), supernatant after centrifugation (S), resin flow through (Ft) and the primary elution fraction (E). After migration on a 12% SDS-PAGE, proteins were transferred on PVDF membrane. Primary antibodies targeting human-IAV PB2 (rabbit IgG) and human-IAV PB1 (mouse IgG) have been used. Revelation was performed with secondary goat antibodies coupled with Alexa Fluor 532 (λ_exi_ = 632 nm, λ_emi_ = 647 nm) and Alexa Fluor 633 (λ_exi_ = 531 nm, λ_emi_ = 554 nm) targeting mouse and rabbit H + L domains respectively, using a Typhoon Trio imaging system (GE Healthcare). After integration of the raw data, PB1 and PB2 revelation are visible in red and green respectively. Black and white signal of PB2 is also shown (bottom) to highlight the PB2 truncations. The upper bands appearing in green/yellow, correspond to unprocessed polyproteins.

Polyproteins are naturally used by certain viruses to structure their proteome^14^,^15^. Recombinant polyproteins mimicking viral polyproteins have recently emerged as a powerful tool to express protein complexes for functional characterisation as well as structural determination (reviewed in^16^). Using this strategy^17^, soluble and active heterotrimeric RNA-dependent RNA-polymerases of influenza B virus (IBV) and bat influenza A (bat-IAV) virus could be produced, crystallized and the structure determined at high resolution^5^,^6^. We have applied a similar strategy to the RdRp of two human-IAV strains, A/Victoria/3/1975(H3N2) and the highly pathogenic A/Viet-Nam/1203/2004(H5N1). The three genes of each heterotrimeric complex were combined with Tobacco Etch Virus (TEV) protease and Cyan Fluorescent Protein (CFP) in a single large open reading frame (ORF). Each gene was separated by a DNA sequence encoding for a peptide segment comprising a short serine/glycine linker and a TEV protease cleavage site (Fig. 1a and supplementary Figure 1). The constructs were optimized for recombinant expression in insect cells using the MultiBac system^18^,^19^. During expression, the TEV protease cleaves the polyprotein co-translationally, resulting in a stoichiometric assembly of the RdRp that can then be selectively purified using an affinity nickel resin. The production of the polyprotein is monitored using the fluorescence of two reporter proteins: CFP encoded within the polyprotein reports directly on recombinant protein yield, while YFP, integrated in the baculovirus genome, monitors virus performance^19^. The ratio of YFP/CFP in our system is a highly useful criterion to determine recombinant polyprotein translation efficacy of the different constructs.

Table 1 summarizes the results obtained with all constructs tested. Several attempts were made to express the H3N2 polymerase heterotrimer (construct number 12, Table 1). Although YFP and CFP can be detected and quantified, CFP values were extremely low compared to YFP with a YFP/CFP ratio of 50, the highest observed for any polyprotein constructs that we expressed. No clear bands were observed on western blot using specific PA, PB1 and PB2 antibodies. Similar results were obtained with the H5N1 RdRp polyprotein, indicating failure of these recombinant expression experiments for these full-length IAV heterotrimeric polymerases. As a comparison, using the same strategy, the YFP/CFP ratio for the expression of IBV or Bat-IAV heterotrimer was close to 20, similar to the ratios we obtained for the expressions of constructs 13 and 15 (i.e. without most of PB2).

In order to understand the difference between the expression of the human-IAV and IBV polymerases, we designed new constructs with a C-terminally truncated PB2 based on available structural information. These were PB2(1–36)^20^, PB2(1–116)^21^ and based on the cap-binding domain^22^, PB2(322–483). Expression and purification experiments were undertaken on constructs 15, 17 and 18, all of which all have full-length PA, PB1 and truncations of PB2 extending to residues 116, 320 and 483 respectively (Fig. 1b). As the PB2 extension increases beyond residue 116, we observed a loss of expression of the polyprotein, thus identifying a critical region in PB2 that limits yield. This can first of all be observed through the monitoring of YFP/CFP reporter genes during expression (Fig. 1c). YFP fluorescence values reach a similar plateau for all constructs whereas CFP values are reduced significantly for constructs 17 and 18. Moreover, after nickel resin pull-down on lysates from an equal amount of cells, we observed by western blot (Fig. 1d) decreased intensity bands of not only PB2 but also PB1, indicative of an overall expression loss. Polyprotein size increase alone could not explain the reduced expression because other control constructs (19 and 20) with additional protein sequences beyond PB2 residue 116 conserve lower YFP/CFP ratios. Furthermore, replacing the PB2(1–116) by another large protein such as RanBP5 (residues 1–1115) generates a construct with a lower YFP/CFP ratio than construct 15. The analysis of the amino acids sequences of PB2 does not provide more indication on the real nature of this phenomenon (supplementary Figure 2).

To determine whether this “loss-of-expression” phenomenon results from our polyprotein expression strategy, which combines PA-PB1 and PB2 in one ORF, we generated a MultiBac baculovirus co-expressing a PA-PB1 fusion and PB2 from an independent expression cassette^23^. This strategy also resulted in high YFP/CFP ratios (≥35), even though the CFP gene was only fused to PA-PB1. These results identify PB2, especially when extended beyond the residue 116, as the bottleneck for expression of complete human-IAV RdRp.

### Dissecting the heterotrimer

**Figure 2:**
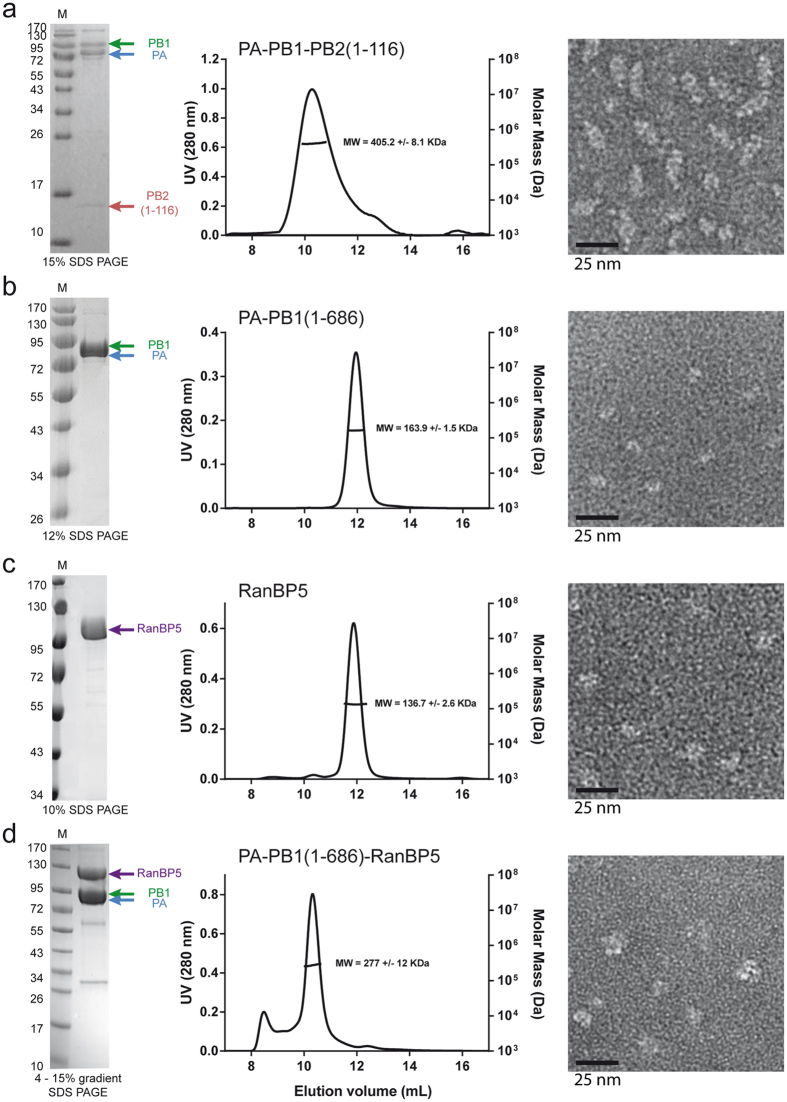
Homogeneous sample analysis of recombinant polymerases, RanBP5 and polymerase-RanBP5. Purified product analysis are horizontally grouped for (**a**) PA-PB1-PB2(1-116), (**b**) PA-PB1(1-686), (**c**) RanBP5 and (**d**) PA-PB1(1-686)-RanBP5. On the left are the Coomassie blue colored SDS PAGE gel of the purified sample with corresponding subunits bands indicated by colored arrows (PA in blue, PB1 in green and RanBP5 in purple). In the middle are the SEC-MALLS chromatograms with the UV signal as a backtrace and molecular weight estimate below the peak. Estimated average molecular weight for each sample is also detailed. SEC-MALLS-RI runs of PA-PB1(1-686), RanBP5 and PA-PB1(1-686)-RanBP5 were performed in the same buffer (50 mM Tris-HCl pH 8.0 and 150 mM NaCl) whereas the SEC-MALLS-RI run of PA-PB1-PB2(1-116) was performed with 50 mM Tris-HCl pH 8.0, 300 mM NaCl and 10% glycerol. (right) corresponding electron microscopy images.

In order to identify a minimal active core of the human-IAV polymerase, we have systematically dissected the heterotrimeric complex for further characterisation using the polyprotein strategy. The expression of PA-PB1 increases significantly when PB2 is totally removed (compare constructs 1 and 13/15; Table 1). The same observation is made after removing the endonuclease domain (i.e. PA-Nter; compare constructs 14 and 13/15). After purification, PA-PB1 forms homogeneous monomeric and stable particles (Fig. 2b) whereas the complexes with PB2 (constructs 15 and 16) give rise to dimers in solution (Fig. 2a). The dimers are stable enough to withstand salt concentrations up to 2 M NaCl during purification, but the SEC elution peaks are broad suggesting that the oligomerization process is quite dynamic. By adding vRNA promoter-like molecules (e.g. the IAV panhandle)^24^, we found that the dimers dissociated into monomeric RNA:protein complexes. We were able to show that the truncated PA-PB1 (construct 1) and the PA-PB1-PB2 (construct 14) exhibit expected polymerase activities. The endonuclease activity is similar to that of the isolated PA-Nter domain (supplementary Figure 3), can be inhibited by the same point mutation (i.e. PA-E80A) or compound (i.e. DPBA) and shows comparable dependency on manganese^25^,^26^. The constructs are also functional in RNA synthesis. Upon addition of a mixture of IAV panhandle, NTPs and [α-^32^P]-UTP, both truncated PA-PB1 and PA-PB1-PB2 are able to generate an 80-nucleotide long product plus other minor products in absence or in presence of ApG (supplementary Figure 4).

We have also shown that further N-terminally truncating PA and/or C-terminally truncating PB1 has no impact on the expression of the corresponding polyproteins (constructs 1 to 11). The heterodimer PA-PB1 can be purified with or without the N-terminal domain of PA (i.e. PA-Nter; compare constructs 1 and 2). However, as soon as the PA-hinge (residues 200–260) is removed, PB1 becomes insoluble. This implies that the PA-hinge linking the Nter- and Cter-domains of PA is crucial for the stability of PB1. However, it is not sufficient in itself since the C-terminal region of PA also interacts with the N-terminal part of PB1^27^,^28^ and cannot be deleted either (compare constructs 2 and 5 to 11). PB1 can be shortened on its C-terminus but when purifying the construct 4 (i.e. PB1 until amino acid 560), only PA-Cter remains soluble. Thus, the minimal construct required to obtain soluble PB1 is construct 3 with PA from 197 to 716 and PB1 from 1 to 660. All the PA-PB1 constructs without the N-terminal domain of PA form dimers in solution (supplementary Figure 5) and adding vRNA-like molecules has no incidence on the dimerization.

Over all the constructs tested, crystals have only been obtained using construct 2 (PA from 197 to 716 and PB1 from 1 to 686). Crystals grew in a few days in low PEG content solutions. They diffract poorly, with diffraction limited to typically ≈ 10 Å.

### PA-PB1 dimer exhibits high-affinity interaction with 5′-vRNA but requires PB2 for binding 3′-vRNA

All influenza RNA segments have the same organization, a central coding region flanked by 2 un-translated regions containing the highly conserved and complementary 5′- and 3′-ends^29^,^30^,^31^. The viral polymerase specifically interacts with both the 5′- and 3′-ends, as recently visualized in crystal structures^5^,^6^, and uses them as a promoter^1^,^2^,^3^. During their biochemical characterisation, we have seen significant effects of vRNA-like RNA molecules on the stability and/or the oligomeric state of most of the constructs. The initial RNA molecule used was the 80-nt panhandle^24^ from which we made the 5′-vRNAp corresponding to the 5′-end (5′-AGUAGAAACAAGGGUA) and it’s 3′ equivalent called 3′-vRNAp (AUACCCUGCUUUUGCU-3′). By thermal shift assay experiments, a 10 °C stabilisation was observed when 5′-vRNAp was added to all the constructs, whereas when 3′-vRNAp was added, the effect was less significant and dependent on the construct. Similar data had already been published^32^.

**Figure 3:**
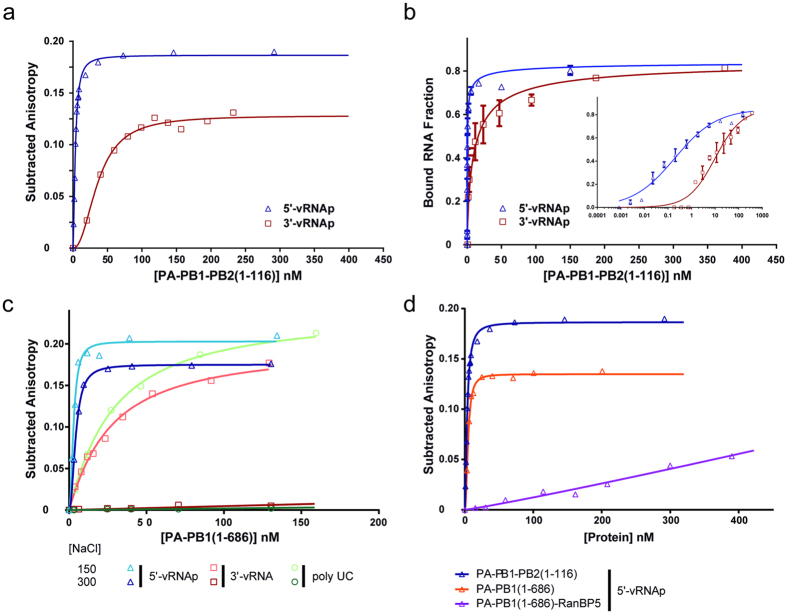
vRNA binding and specificity. (**a**) Binding titration of the truncated trimer PA-PB1-PB2(1-116) towards the 5′-vRNAp (blue triangle) and 3′-vRNAp (red square) sequences using fluorescence anisotropy at 300 mM NaCl. (**b**) Binding titration performed by filter binding assay against P^32^ labelled 5′-vRNAp (blue triangle) and 3′-vRNAp (red square) using 300 mM NaCl. Bound RNA fraction is plotted as a function of polymerase concentration. (**c**) Binding titration of the truncated dimer PA-PB1(1-686) performed at 150 and 300 mM NaCl against the 5-′vRNAp (dark and light blue triangles), 3′-vRNAp (orange and dark red squares) and polyUC RNA (light and dark green circles) by fluorescence anisotropy. (**d**) Binding titration of different polymerases and polymerase-RanBP5 constructs against the 5′-vRNAp at 300 mM NaCl by fluorescence anisotropy. PA-PB1-PB2(116) and PA-PB1(1-686) are depicted by blue and orange triangles respectively, PA-PB1(1-686)-RanBP5 is depicted with purple triangles. For all anisotropy titrations (**a**,**c**,**d**) subtracted anisotropy is plotted as a function of protein concentration.

We used fluorescence anisotropy to measure the interaction between our truncated polymerase constructs and the conserved vRNA ends by following the fluorescence polarization increase of a fluorescently labelled RNA when it binds the polymerase. For this purpose, we used 5′-vRNAp and 3′-vRNAp, both labelled with fluorescein amidite (FAM) at the opposite extremity of the putative interaction, i.e. at the 3′-end for the 5′-vRNAp and *vice versa* (Fig. 3 and Table 2). At 300 mM NaCl, the Kd for the 5′-vRNAp are similar (sub-nanomolar) for both constructs 1 and 14, whereas for the 3′-vRNAp, a Kd of 36 nM for the construct with PB2 is obtained (Fig. 3a) and no binding was observed for the construct without PB2 (Fig. 3c). As the estimated Kd for the 5′-end was in a range below the working concentration and beyond the sensitivity limit of the fluorescence anisotropy measurement, another method was required to determine the 5′-vRNAp affinity. Filter binding assays (FBA) were then therefore used for precise Kd determination (Fig. 3b). With the corresponding radioactive probes, we found values of 0.2–0.4 nM for the 5′-end. For the 3′-end, the Kd was 100-fold higher and critically dependent on the presence of PB2. Salt concentration is also an important parameter. We show different binding characteristics at 150 and 300 mM NaCl of the PA-PB1 towards different RNAs. The Kd value of PA-PB1 for the 5′-end remains sub-nanomolar in the range of 150 to 300 mM NaCl. In contrast, at 150 mM NaCl, 3′-vRNAp binds to PA-PB1 with the same affinity than for a poly-UC (Fig. 3c), and so is non-specific. All relevant titrations were performed at 300 mM NaCl to prevent non-specific affinity measurements. Recently published affinity data^33^ working at 500 mM NaCl likewise consistent with very high affinity of the 5′-vRNAp towards IAV polymerase but this study failed to measure 3′-vRNAp binding within the nanomolar range, indicating that 3′-vRNAp binding is also tightly influenced by salt concentration.

### RanBP5 interacts tightly with PA-PB1(1-686) when co-expressed

During the viral cycle, the assembly of influenza RdRp follows a multi-factorial pathway involving many host partners proteins. After transcription in the nucleus, viral mRNAs are exported to the cytoplasm to be translated by the cellular machinery. The components of the replication machinery (i.e. PA, PB1, PB2 and NP) then must be imported into the nucleus. Whereas PB2 and NP use importins-α^9^,^34^,^35^,^36^,^37^, PA and PB1 are conjointly imported as a preformed heterodimeric submodule by the importin-β RanBP5^10^,^12^,^38^. Having confirmed the existence of a stable PA-PB1 complex we sought to demonstrate its interaction with RanPB5. The human *IPO5* gene which encodes for RanBP5, was expressed in insect cells. After purification, RanBP5 forms a homogeneous monomeric sample (Fig. 2c) that has been used in mixing experiments with freshly purified PA-PB1(1-686) followed by SEC-MALLS-RI experiments. The two samples were mixed in stoichiometric ratio and incubated before size exclusion chromatography (SEC). Both RanBP5 and PA-PB1(1-686) were eluted in the same single peak, but MALLS-RI indicated a molecular weight corresponding of a mixture rather than a stable ternary complex (supplementary Figure 6). We then attempted to produce a PA-PB1(1-686)-RanBP5 heterotrimer by the self-processing polyprotein strategy. A long ORF encoding for the trimeric complex was created by inserting the *IPO5* gene downstream of the PB1(1-686) coding sequence (Table 1, construct 21). Remarkably, from this polyprotein construct, PA-PB1 co-purifies with RanBP5, forming a homogeneous and stable heterotrimer (Fig. 2d).

**Figure 4:**
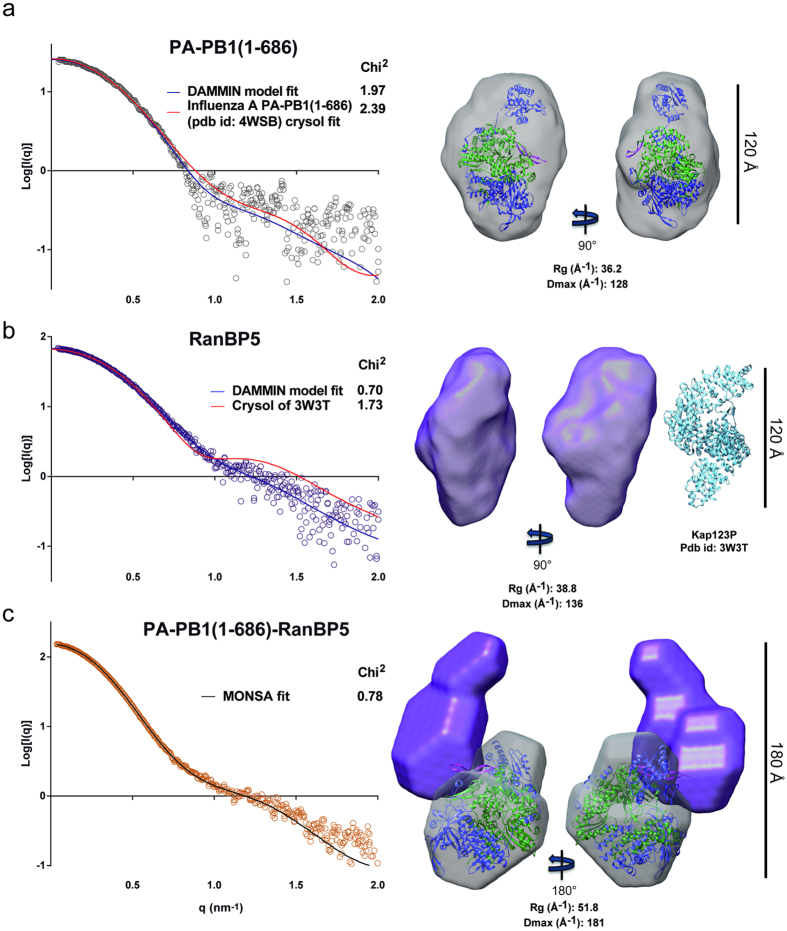
Online SAXS analysis of PA-PB1, RanBP5 and PA-PB1-RanBP5 complex in solution. Results are shown for (**a**) PA-PB1(1-686), (**b**) RanBP5 and (**c**) PA-PB1-RanBP5. On the left are the experimental Ln[I(q)] curves with the *ab-initio* DAMMIN curve fit (blue) using the Damstart (DAMAVER) as an initial constraint and the CRYSOL fit (red) of the closest homologous X-ray structure. The MONSA fit is also shown for PA-PB1-RanBP5 in black. The Chi^2^, to evaluate the statistical similarity between experimental intensities and those computed from a model, are also shown. On the right, the homologous PDB structure of PA-PB1(1-686) from Bat-IAV polymerase is depicted in cartoon and fitted in both the DAMAVER and the MONSA generated envelops of PA-PB1(1-686) which are depicted in grey. PA is coloured in blue, PB1 in green and the NLS of PB1 in magenta. The DAMAVER and MONSA envelop of RanBp5 is coloured in purple. As a comparison, the atomic structure of Kap123P (pdb is: 3W3T) is depicted in cartoon with a sky blue colour alongside the DAMAVER envelop of RanBP5. All envelopes and models are adjusted at the same size scale.

Online SEC-SAXS was used for the characterisation of PA-PB1(1-686), RanBP5 and PA-PB1(1-686)-RanBP5, respectively in solution. Using the Vc determination method^39^ on the diffusion data of PA-PB1(1-686) shows a Mw estimate of 146 kDa (Table 3 and supplementary Figure 7). The calculated Mw is 166 kDa. The Guinier transform measures a hydrodynamic radius (Rg) of 36.2 Å and GNOM produces the pair distribution function fit with a Dmax of 128 Å. Further analysis of the SAXS curve (Fig. 4a) shows a visually adequate correlation with the CRYSOL curve which uses the bat-IAV polymerase structure (PDB id: 4WSB) as a model. To evaluate the statistical similarity between experimental intensities and those computed from a model has been derived. The high chi^2^ values are observed for this dataset, due to a noisy SAXS curve. A slight deviation of the fit is observed above a q range of 0.8 nm^−1^, suggesting conformational differences between the scattering curve and the crystal structure coordinates. *Ab-initio* modelling was performed using 15 DAMMIF models which were averaged by DAMAVER. Averaged model correlation with the diffusion curve was then checked using the damstart file as a starting envelope for DAMMIN. The polymerase DAMAVER envelope (Fig. 4a) appears as a pear shaped structure in which the homologous model can be fitted. Additional envelope volume is visible close to the endonuclease domain, implying that it adopts multiple conformations in solution.

Using the same methodology, we determine RanBP5 to be a monomer in solution with a measured Mw of 144 kDa close to the theoretical Mw of 126 kDa (Table 3 and supplementary Figure 7). RanBP5 displays an Rg of 38.8 Å and a Dmax of 136 Å. With a Mw lower than that of PA-PB1(1-686), RanBP5 displays a larger Dmax and Rg indicating an extended structure. We have looked over the *Protein Data Base* (PDB) and found yeast karyopherin Kap121^40^ to be the closest homologue available for RanBP5 (30% sequence identity over 1027 residues). CRYSOL curve fitting using the apo form of Kap121 (PDB id: 3W3T) against the diffusion curve reveals important structural and conformational differences between the two structures (Fig. 4b). However, envelope modelling confirms an elongated structure of RanBP5 in solution, comparable in size with yeast Kap121 in the crystal structure.

Analysis of PA-PB1(1-686)-RanBP5 describes a significantly larger complex in solution with an Rg of 51.8 Å and a Dmax of 181 Å. The Mw estimate of 323 KDa is close to the expected Mw of 292 KDa, confirming the presence of a stoichiometric 1:1:1 PA-PB1(1-686)-RanBP5 complex in solution. MONSA builds *ab initio* shapes using the scattering curves for complexes together with those of their individual components^41^. MONSA was used to perform the *ab-initio* modelling of the complex PA-PB1(1-686)-RanBP5 (supplementary Figure 8). It separately uses the PA-PB1(1-686) and RanBP5 diffusion curves (Fig. 4a,b) in combination with that of PA-PB1(1-686)-RanBP5 to propose a consensus dual envelope (Fig. 4c). Model fitting was performed against the three experimental curves and matches all 3 curves with similar quality. With this *ab-initio* modelling we also find a comparable envelope for PA-PB1(1-686) like the one produced with DAMMIF/DAMAVER. RanBP5 on the other hand displays a different envelope suggesting conformational differences between the unbound and bound forms of RanBP5. The model suggests a close interaction between the two proteins, RanBP5 (violet envelope) interacts with PA-PB1(1-686) (grey envelope), suggesting molecular contacts well beyond those of the proposed PB1 nuclear localization signal (NLS) containing domain, which the crystal structure shows is a mobile and solvent exposed β-ribbon^6^. This model further supports the hypothesis that RanBP5 may play a chaperone function for PA-PB1 prior to assembly with PB2 in the nucleus to complete the polymerase.

### RanBP5 regulates the binding of the 5′-vRNAp

The PA-PB1 complex not only exists as a stable submodule but can moreover recognise the 5′-vRNAp with a high affinity and acts as a replicase and endonuclease on its own, without PB2 present. The current model predicts the NLS of PA-PB1 to be present within PB1 (residues 187–211)^12^, at the end of a beta-strand located parallel to the vRNAp binding sites, indicating a potential link between the binding to RanBP5 and/or the binding to vRNAp.

Subsequently to measuring the sub-nanomolar Kd of PA-PB1(1-686) and PA-PB1-PB2(1-116) to the 5′-vRNAp, an identical experiment was performed with the PA-PB1(1-686)-RanBP5 complex (Fig. 3d). The association curve clearly shows a complete loss of binding affinity towards the 5′-vRNAp when RanBP5 is present, both at 300 mM and 150 mM NaCl. Using SEC-MALLS-RI experiments, we have shown that an excess of 5′-vRNAp does not induce any complex dissociation of the PA-PB1(1-686)-RanBP5 trimer (supplementary Figure 9). Taken together, these experiments suggest that RanBP5 binding obscures the 5′-vRNAp binding site, thus blocking specific 5′-vRNAp binding.

## Discussion

Expression of full-length IBV and bat-IAV polymerases has been successfully achieved using a polyprotein approach, resulting in atomic resolution structures by X-ray crystallography^5^,^6^. In marked contrast and unexpectedly, efficient expression of active full-length human or avian IAV polymerase at yields high enough for structural studies has not been reported to date, irrespective of the method used. Our studies identify PB2, and notably residues 120 to 250 as responsible for this poor expression. Interestingly, attempts by a different research group to reconstitute a full IAV polymerase (e.g. full IAV polymerase expression/purification for structural studies) likewise failed beyond the N-terminal PB1 interacting domain of PB2^13^. Furthermore, the recent bat-IAV, IBV and ICV RdRp crystal structures have shown that PB2 can adopt a plethora of conformations, demonstrating how dynamic PB2 is within the heterotrimer^5^,^6^,^7^,^42^,^43^. The reason why PB2 hinders polyprotein expression is not clear at the moment. We speculate that the extended fold of the PB2 region 1–250 is unstable unless bound to one side of PB1^5^,^6^ and thus may require chaperones to maintain its stability prior to assembly onto PA-PB1. Of note, Hsp90 has been shown to stimulate influenza virus RNA synthesis^44^ and the nuclear import of the subunits^35^. Moreover, Hsp90 inhibitors reduce influenza virus replication in cell culture^45^. We have shown previously that provision of co-factors in *trans* can ameliorate the quality and quantity of target proteins expressed in the baculovirus/insect cell system^46^,^47^. Provision of Hsp90 and the other chaperones, within the polyprotein or in *trans* on the baculovirus backbone, may provide a powerful handle to overcome the bottleneck of human-AIV polymerase production. Furthermore, although the cellular context between viral infection and the expression of recombinant proteins is different, it cannot be excluded that the polyprotein interferes with the cellular innate immunity. IAV RdRp, and PB2 in particular, has been shown to regulate host anti-viral response through the binding to IPS-1 and the inhibition of interferon β production^48^.

PA-PB1 can be produced as a stable submodule and forms a discrete heterodimer in solution. Dimerization of PA-PB1 heterodimers can appear when either the endonuclease is removed or the N-terminal of PB2 is added which may indicate that the superstructure formation could be artificially induced by truncations of the full-length polymerase. PA-PB1 can specifically bind to the 5′-vRNAp with sub-nanomolar affinity in solution. In contrast, 3′-vRNAp binding requires the presence of PB2 and is within the low nanomolar range, indicating a much stronger preference of the polymerase towards the 5′-vRNAp. These results are consistent with the high-resolution crystal structures in which the 5′-vRNAp binding site is located between the PA and PB1 subunits^5^,^6^. The 3′-vRNA, on the other hand, interacts with all three subunits suggesting a sequential vRNAp binding mechanism.

Our results corroborate key steps of influenza polymerase assembly^10^,^38^,^49^. Purification of stoichiometric complexes using the polyprotein strategy confirms that PA-PB1 can exist as a preformed and stable submodule, independently of additional host factors, with PA acting as a putative “chaperone” for PB1. Our data also shows that PA-PB1 is functional in RNA synthesis, which has interesting physiological implications. Moreover, we demonstrate here that the PA-PB1(1-686) heterodimer can assemble with RanBP5 into a stable, heterotrimeric complex that can be purified *in vitro*. This likely corresponds to the import complex for these two subunits, although other chaperones such as HSP90 may be co-imported^35^. In contrast to PA-PB1 with RanBP5, we failed to reconstitute a binary PB2-importin-α complex using the polyprotein strategy even though functional and structural data show that PB2 nuclear import depends on the importin-α pathway, through a direct interaction between the PB2 C-terminal domain that contains the NLS^8^,^9^. This is probably partly due to an inherent instability of isolated IAV PB2 in insect cells but could also reflect the lack of sufficient chaperones, co-chaperones or other unidentified factors that stabilise PB2. Furthermore, cytoplasmic expression of PB2 appears to destabilise many cellular processes^50^,^51^. We also still do not know the reason for the difference in the import pathway usage between the PA-PB1 submodule and PB2. The lack of high expression levels of fully assembled PA-PB1-PB2 heterotrimers unfortunately currently limits our efforts to elucidate the structure of the complete human IAV polymerase at atomic resolution. Nevertheless, docking of bat-IAV and IBV crystal structures into the PA-PB1(1-686)-RanBP5 SAXS envelope suggests that assembly of PB2 onto the PA-PB1-RanBP5 complex might be possible, based on steric considerations. Indeed RanBP5 could even facilitate trimer assembly perhaps concomitantly with Ran-dependent dissociation from PA-PB1. In contrast, RanBP5 profoundly affects the ability of PA-PB1 to bind vRNA. We show that whereas the 5′-vRNA is specifically recognized by PA-PB1 with high affinity, this is abolished in the ternary complex with RanBP5. This again suggests that RanBP5 disassociation may be coupled to assembly of the polymerase with the promoter RNA. In any case, only when PB2 associates is the polymerase able to associate with the 3′-vRNA end, consistent with the crystal structures^5^,^6^. But surprisingly, the affinity of the heterotrimer for 3′-vRNA is in the same order of magnitude than the affinity of NP for RNA substrates (Table 2), revealing a potential competition between the complete polymerase heterotrimer and the nucleoprotein.

We demonstrate here for the first time that the polyprotein expression strategy can be used not only to co-express subunits that form a stable complex, but also to successfully reconstitute interactions with downstream partner proteins *in vivo* in the expression host for direct purification and functional characterisation. Functional studies on IAV RdRp reveal that vRNAp promoter binding is completely abolished when PA-PB1(1-686) is bound to RanBP5, suggesting a possible role for RanBP5 in maintaining PA-PB1 in an inactive form during nuclear import. This discovery holds a promise for influenza research as it provides a novel point of therapeutic intervention for targeting and inhibiting influenza virus during assembly of its active components. We anticipate that compounds which block polymerase/RanBP5 binding or release may prove to be highly efficient, broad spectrum antiviral inhibitors in the treatment of influenza, in particular for patient groups where classical vaccination strategies fail.

## Methods

### Molecular biology

To avoid expression of alternative proteins, all the genes used for this work are synthetic genes, codon optimised for insect cells expression. The DNA coding sequences of A/Victoria/3/1975(H3N2), the highly pathogenic A/Viet-Nam/1203/2004(H5N1) polymerase subunits and human *IPO5* have been ordered to GeneArt (ThermoFisher Scientific), optimized for the expression in insect cells. Cloning has been achieved following the supplier procedures (New England Biolabs). The pPBAC plasmid was used for the polyprotein constructs^16^,^18^. Few constructs have been expressed using pFastBac-HTB (Life Technologies). vRNA-like molecules (i.e. IAV panhandle)^24^ have been produced and purified using classical *in vitro* transcription protocols^52^,^53^.

### Expression and purification

Large scale suspension cultures expressing polymerase fusion constructs were prepared using High Five insect cells grown in Express Five media (Life Technologies) at 0.5 × 10^6^ cells/mL infected at 0.2% (V/V) with the baculovirus mother solution. Cultures were maintained at 0.5–1 × 10^6^ cells/mL until proliferation arrest (24–48 h after infection). Following the proliferation arrest YFP and CFP measurements were performed every 12 h until a fluorescence plateau was reached (72–96 h after infection). Cultures were then spun down at 800 g for 10 min and cell pellets were stored at −80 °C.

Cell pellets were resuspended in 50 mL of lysis buffer (50 mM Tris-HCl pH 8.5, 300 mM NaCl and 2 mM β-mercaptoethanol) per 500 × 10^6^ cells in the presence of EDTA-free anti-protease cocktail (complete from Roche). Lysis was performed with two cycles of freezing (−180 °C)/thawing (26 °C) after which 10% of glycerol were added to the lysate before centrifugation (45 min, 40 000 g, 4 °C). After retrieval of the clarified lysate, 30 mM of imidazole pH 8.0 were added before loading on Ni-NTa superpose resin (Quiagen). After flowing the lysate through the resin, three wash steps with 10 column volumes (CV) of buffer A (lysis buffer with 30 mM imidazole), 10 CV of wash buffer B (50 mM Tris-HCl pH 8.5, 1 M NaCl, 10% glycerol and 2 mM β-mercaptoethanol) and 10 CV of wash buffer A were performed. Elution of the bound complex was performed with 300 mM imidazole. Elution fractions containing polymerase were then pooled together and directly injected on a 5 mL Hitrap heparin resin (GE healthcare) which had previously been equilibrated with 5 CV of buffer A. After binding to the resin, a 5 CV wash was performed with buffer A before eluting with a 40 mL salt gradient on a FPLC system. The elution peak corresponding to stoichiometric polymerase assemblies was then pooled and injected on an S200 (GE healthcare) size exclusion chromatography equilibrated in 50 mM Tris-HCl pH 8.5, 300 mM NaCl, 5 mM β-mercaptoethanol. Peak fractions were pooled and concentrated to the desired concentration using a 100 kDa concentrator. Once concentrated the protein prep could be stored by addition of 20% glycerol and flash freezing in liquid nitrogen.

NP was purified using the protocol described in^54^,^55^.

### SEC-MALLS-RI analysis

All MALLS runs were performed using a S200 increase SEC column (10/300 GL, GE Healthcare). Sample injection and buffer flow was controlled by a Hitachi L2130 pump, following the SEC column was a L-2400 UV detector (Hitachi), Optilab T-rEX refractometer (Wyatt technologies) and a DAWN HELEOS-II multi angle light scattering detector (Wyatt technologies). Prior to injection, columns and systems were equilibrated in 5 to 10 column volumes of running buffer. 50 μL injections were performed using protein samples concentrated at a minimum of 2 mg.mL^−1^, constant flow rate of 0.5 mL.min^−1^ was used. Accurate MALLS mass prediction was performed with the Astra software (Wyatt Technologies). Curves were represented with Graphpad (Prism).

### Electron microscopy

Samples (0.1 mg.mL^−1^) were applied to the clean side of carbon on mica. The carbon was then floated on sodium silicotungstate (2% v/w) or uranyl acetate (2% v/w) and a grid placed on the top of it. After air-drying, the samples were observed in a T12 FEI electron microscope and images were taken using an Orius SC1000 CCD camera (Gtan Inc., Pleasanton, CA).

### SAXS analysis

All datasets were collected on BM29 (ESRF). The initial trials using direct sample measurement showed a slight concentration dependency of the Guinier estimated Rg. Online SEC-SAXS was therefore used for all experiments in order to limit the contribution of partially aggregated, oligomeric or dissociated protein to the experimental curve. The experimental setup consists of a High Pressure Liquid Chromatography (HPLC) system connected to an analytical S200 increase column (5/150 GL, GE Healthcare) followed downstream by the SAXS sample capillary. SAXS measurements were performed every second with a Pilatus 1 M detector at distance of 2.87 m allowing a q range of 0.03 to 4.5 nm with a wavelength of 0.01 nm.

Following data collection, experimental curves were subtracted and analysed using Primus (ATSAS) or Scatter (Bioisis) programs suits^56^. To verify the molecular mass, the Rambo and Tainer method was used^39^,^57^. Rg predictions using the Guinier extrapolation were plotted against the elution volume to select the most monodisperse part of the protein elution peak. SAXS datasets within this zone were then scaled and averaged to produce one unique I(q) curve. GNOM^58^ was then used to produce a P(r) function which was subsequently used by DAMMIF^59^ to generate 15 *ab-initio* models. DAMAVER^60^ was then used to generate an average model. Consistency of the DAMAVER averaging with the original experimental curve was assessed by using DAMMIN^41^ and using the damstart.pdb envelope as a starting model. Homologue PDB structure comparison was assed using Crysol^61^. Multiple diffusion curve *ab-initio* modelling was performed using Bunch^62^. Homologue structure fitting within the DAMAVER envelope was performed with Chimera^63^ and curve representations using Graphpad (Prism).

### Fluorescence anisotropy assay

Equilibrium RNA binding experiments were performed with the following vRNA set synthetized by Integrated DNA Technologies (IDT): 5′-vRNAp (5′-pAGUAGAAACAAGGGUA-FAM3′), 3′-vRNAp (5′FAM-AUACCCUGCUUUUGCU-3′) and polyUC (5′-pUCUCUCUCUCUCUCUCUC-FAM3′). 5′-vRNA sequences were synthetized with a 5′-mono phosphate and labeled with 6-FAM in 3′OH. 3′ vRNA were labeled with FAM in 5′. Polymerase constructs was titrated into fluorescently labeled RNA using the buffer (50 mM HEPES pH 7.5, 5 mM β-mercaptoethanol plus 150 mM or 300 mM NaCl) at room temperature. Initial reactions were performed with 2–4 nM of RNA with an initial volume of 600 μL to limit the dilution effect of protein addition (<5%). Anisotropy measurements were undertaken using a PTI fluorometer equipped with automated polarizers. The excitation and emission wavelengths were 494 and 521 nm, respectively. 50 anisotropy measurements with a 1 second integration time were performed per titration point.

### Filter Binding Assay

Equilibrium RNA binding experiments were undertaken with the same vRNA set synthetized by IDT. vRNA were labeled with^32^ P in 5′ using the T4 polynucleotide kinase (New England Biolabs). Double filter binding was used following the previously established protocol^64^. Protein concentration was titrated against constant concentrations of vRNA (≤0,01 nM) within a final volume of 200 μL using the standard protein buffer (50 mM HEPES pH 7.5, 300 mM NaCl, 5 mM β-mercaptoethanol). 180 μL were then filtered on two membranes using a 96 well Whatman Minifold dot-blot apparatus. The first membrane was Protran BA 85 membrane (Whatman) to retain the polymerase and the second one is a nylon Hybond-N + membrane (Amersham Bioscience) to retain unbound nucleic acid. Both membranes were pre-incubated 1 h at room temperature within the protein buffer prior to use. After blotting, another 180 μL of protein buffer were run through the membranes to wash any unbound RNA before letting the vacuum dry the dot-blots for 30 seconds. Both membranes were then dismounted from the dot-blot apparatus and exposed to a storage phosphor screen (BAS storage phosphor screen, GE Healthcare) overnight. Revelation of the phosphor screen was then performed with a Typhoon Trio imaging system (GE Healthcare). Dot blot phosphorescence intensity was integrated using the Image J software. A derived bound ratio was then calculated using the following formula (1):


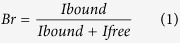


Where I_bound_ is the integrated intensity of protein/RNA complex which is retained by the protran membrane and I_free_ is the unbound RNA retained by the nylon Hybond-N + membrane.

### Binding assay data analysis

RNA binding curves were plotted with the subtracted anisotropy or Bound RNA ratio as a function of the protein concentration. Sigmoidal binding curves were fitted to the data using GraphPad (Prism) with the following two equations:

(1) For the anisotropy titrations, [vRNA] ≈ Kd. Assuming a 1/1 stoichiometry the following equation (2) was used to estimate the Kd


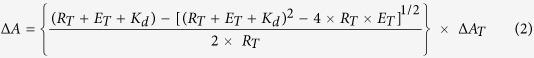


Where ∆A is the change in subtracted anisotropy, *∆A*_*T*_ the total change of subtracted anisotropy. *R*_*T*_ is the total vRNA concentration, *E*_*T*_ is the total polymerase concentration at each given point and Kd is the dissociation constant.

(2) In the case of filter binding assay, a single site isotherm was used as we consider that [vRNA] ≪ Kd. This equation (3) therefor assumes an effective equality between the free polymerase concentration and the total polymerase concentration.


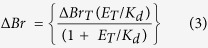


Where *∆Br* is the change in binding ratio, *∆Br*_*T*_ is the total change in binding ratio. *E*_*T*_ is the total polymerase concentration at every point in titration and Kd is the dissociation constant.

Standard error calculations for the apparent Kd within a given condition were calculated through triplicate data measurement.

## Additional Information

**How to cite this article**: Swale, C. *et al.* Structural characterization of recombinant IAV polymerase reveals a stable complex between viral PA-PB1 heterodimer and host RanBP5. *Sci. Rep.* **6**, 24727; doi: 10.1038/srep24727 (2016).

## Supplementary Material

Supplementary Information

## Figures and Tables

**Table 1:** Details of human-IAV RdRp polyproteins.

	Construct number	PA	PB1	PB2	RanBP5	YFP/CFP	Duplicate in H5N1	Solubility
heterodimers	1	1–716	1–686	–	–	6	✓	yes
	2	197–716	1–686	–	–	4	✓	yes
	3	197–716	1–660	–	–	4	✓	yes
	4	197–716	1–560	–	–	4	✓	no
	5	210–716	1–686	–	–	6	✓	no
	6	222–716	1–686	–	–	6	✓	no
	7	231–716	1–686	–	–	6	✓	no
	8	240–716	1–686	–	–	6	✓	no
	9	250–716	1–686	–	–	6	✓	no
	10	263–716	1–686	–	–	6	✓	no
	11	197–263	16–686	–	–	n.d.*	✓	no
heterotrimers	12	1–716	1–757	1–759	–	50	✓	n.d.
	13	1–716	1–757	1–36	–	15	✓	yes
	14	197–716	1–757	1–116	–	7	✓	yes
	15	1–716	1–757	1–116	–	17	✓	yes
	16	1–716	1–757	1–250	–	28	✓	n.d.
	17	1–716	1–757	1–320	–	30	✓	n.d.
	18	1–716	1–757	1–483	–	41	✓	n.d.
	19	1–716	1–757	1–116 – MBP	–	17	✓	yes
	20	1–716	1–757	1–116 – 320–483	–	20	✓	yes
	21	1–716	1–686	–	1–1115	12	–	yes

The expressions have been made on both A/Victoria/3/1975(H3N2) and A/Viet-Nam/1203/2004(H5N1) strains. Identical results were obtained with the 2 strains. The table provides the data obtained with the A/Victoria/3/1975(H3N2) strains. The ratio YFP/CFP were calculated with the 2 maxima of the fluorescence spectra measured on the same sample (YFP: λ_exi_ = 488 nm, λ_emi_ = 525 nm ; CFP: λ_exi_ = 430 nm, λ_emi_ = 480 nm) corresponding to 1 × 10^6^ infected cells. This ratio is proportional to the level of expression of the corresponding constructs. The table contains also data on the solubility of each construct in the classical purification buffer (i.e.: 50 mM Tris-HCl pH 8.5, 300 mM NaCl, 2 mM β-mercaptoethanol, 2–10% glycerol). *This construct has been cloned in pFastBac-HTB without TEV nor CFP. The only fluorescent reporter protein was the YFP. Its signal was in accordance with the YFP values obtained for the other constructs.

**Table 2:** Titration measurements against vRNA promoters.

Construct	NaCl (mM)	RNA	Kd (nM)	std error (nM)	R^2^
construct **14**:PA-PB1-PB2(1-116)		5′-vRNAp	0.83 (0.19)	0.08 (0.02)	0.99 (0.97)
	300	3′-vRNAp	36 (17)	2 (2)	0.99 (0.95)
		polyUC	≥1000	n.d.	n.d.
construct **1**:PA-PB1(1-686)		5′-vRNAp	0.87 (0.38)	0.1 (0.05)	0.99 (0.99)
	300	3′-vRNAp	≥1000	n.d.	n.d.
		polyUC	≥1000	n.d.	n.d.
		5′-vRNAp	0.53	0.12	0.99
	150	3′-vRNAp	22.1	1.0	0.99
		polyUC	22.8	0.7	0.99
construct **21**:PA-PB1(1-686) RanBP5	300	5′-vRNAp	≥1000	n.d.	n.d.
NP	300	polyUC	29	1.2	0.94

Values in parentheses correspond to the values obtained by filter-binding assay experiments. All the experiments have been made in triplicate.

**Table 3:** SAXS data-collection and scattering-derived parameters.

	construct 1:PA-PB1(1-686)	construct 21:PA-PB1(1-686)-RanBP5	construct 22 :RanBP5
Data collection parameters			
Instrument	ESRF - BM29		
Beam size at sample (μm)	700 × 700		
Wavelength (Å)	0.9919		
*q* range (Å^−1^)	0.25–50		
Detector	Pilatus 1 M		
Detector distance (m)	2.867		
Exposure (s per image)	1		
Column	S200inc 5/150 GL		
Flow rate (mL.min^−1^)	0.5	0.4	0.5
Injected sample concentrations (mg.mL^−1^)	3.4	4.3	8.5
Injection volume (μL)	50		
Temperature (K)	293		
Structural parameters			
R_g_ (Å) [from *P*(*r*)]	37.9 ± 0.1	52.2 ± 0.1	39.4 ± 0.2
R_g_ (Å) [from Guinier]	36.2 ± 0.4	51.8 ± 0.5	38.8 ± 0.8
*D*_max_ (Å)	128	181	136
Porod volume estimate (Å^3^)	254 630	577 070	215 890
Molecular-mass determination			
Molecular mass *M*_r_ (Da) [from Rambo]	146 493	323 311	143 827
Calculated *M*_r_ (Da) from sequence	165 915	291 983	125 892
Software employed			
Primary data reduction	PRIMUS		
Data processing	PRIMUS		
*Ab initio* analysis	DAMMIF		
Validation and averaging	DAMAVER & DAMMIN/MONSA		
Computation of model intensities	CRYSOL		
3D graphics representations	CHIMERA		
