# Supplementary material for: Structural characterization of recombinant IAV polymerase reveals a stable complex between viral PA-PB1 heterodimer and host RanBP5
Source: Sci Rep. 2016 Apr 20;6:24727. doi: 10.1038/srep24727 (PMC4837377; doi:10.1038/srep24727)

Structural characterization of recombinant IAV polymerase reveals a stable complex between viral PA-PB1 heterodimer and host RanBP5

Christopher SWALE[1,2,3$], Alexandre MONOD[1,2$], Laura TENGO[1,2,3], Alice LABARONNE[1,2,3], Frédéric GARZONI[1,2,4], Jean-Marie BOURHIS[1,2,3], Stephen CUSACK[1,2,4], Guy SCHOEHN[1,2,3], Imre BERGER[1,2,4,5], Rob WH RUIGROK[1,2,3] & Thibaut CREPIN[1,2,3*]

Supplementary information

Supplementary Figure 1

Sequences of the main polyprotein constructs. The figure details the open reading frames of constructs 12, 15 and 21. The accession numbers for the protein sequences of the TEV protease, the A/Victoria/3/1975(H3N2) and A/Viet-Nam/1203/2004(H5N1) RdRP subunits, human RanBP5 isoform 3 and the cyan fluorescent protein are provided in the right part of the schema. The amino acid sequences for the TEV-cleavage site and the polyhistidine affinity tag are coloured respectively in green and red. All the accession numbers correspond to UniProtKB access numbers, except the sequence for the TEV protease (NCBI accession number).

Supplementary Figure 2

Sequences alignment of PB2. The alignment has been made for the sequences of the 2 human-IAV strains used for this work plus the 2 strains for which the structures have been recently solved. bat-IAV corresponds to A/little yellow-shouldered bat/Guatemala/060/2010(H17N10) (UniProtKB access number: H6QM90), H3N2-IAV to A/Victoria/3/1975(H3N2) (H9XIJ5), H5N1-IAV to the highly pathogenic A/Viet-Nam/1203/2004(H5N1) (Q6DNN3) and IBV to B/Memphis/13/2003 (Q5V8X3). The secondary sequences elements shown over and below the sequences alignment correspond to bat-IAV (PDB access numbers 4WSB) and IBV (4WSA) respectively. The alignment has been done using Clustal X2 [1] and drawn with ESPript [2].

Supplementary Figure 3

Endonuclease activity. An unstructured 52 nt poly-UC RNA (10 µM) [3] was incubated with the wild-type (wt) or E80A mutant of PA-PB1-PB2(1-116) (12 µM) or the N-terminal domain of PA (PA-Nter) (12 µM) for 2 h at 37°C in a final volume of 30 µl. Reactions were done in the presence and/or in the absence of 0.5 mM $MnCl_2$ in the reaction buffer 50 mM Tris-HCl pH 8,5, 25 mM KCl, 2,5 mM NaCl, 10

mM β-mercaptoethanol as previously described [3,4]. Reactions were inhibited by addition of 2 mM 2,4-dioxo-4-phenylbutanoic acid (DPBA) in the same conditions. As controls, the RNA was incubated with 0.5 mM $MnCl_2$ or with 2 mM DPBA or alone (ctrl). Reactions were stopped by adding 20 mM EGTA. The reactions were then loaded on an 8 M urea polyacrylamide gel. After migration, the gel was revealed by methylene blue staining.

Supplementary Figure 4

***De novo* synthesis activity assay.** (**a**) PA-PB1(1-686) and (**b**) PA-PB1-PB2(1-116) polymerases were incubated at 37°C with the 80-mer panhandle RNA, NTPs and radiolabelled [αP^{32}] UTP. Reactions were stopped at 30, 60, 90 and 120 minutes with the addition of EGTA before loading on a 15 % urea PAGE. Revelation was performed using a phosphorus screen and Typhoon scanner (GE Healthcare).

Supplementary Figure 5

Effect of PA-Nter depletion on PA-PB1 oligomerzation. Individual SEC-MALLS chromatography runs of (**a**) PA-PB1(1-686) and (**b**) PA(196-716)-PB1(1-686) have been performed in the same buffer (50 mM Tris-HCl pH 8.0, 150 mM NaCl, 5 mM β-mercaptoethanol) using a S200increase (10/300 GL, GE Healthcare) column. UV absorbance at 280 mm is plotted against the elution volume. MALLS-RI molecular mass estimates are plotted below the curve.

Supplementary Figure 6

RanBP5 and PA-PB1(1-686) does not form a stable complex when mixed. PA-PB1(1-686) and RanBP5 were separately purified before being mixed in equimolar proportions. The mixture was incubated 1h at 20°C before analysis. Individual SEC-MALLS chromatography runs of (**a**) PA-PB1(1-686), (**b**) RanBP5 and (**c**) PA-PB1(1-686) + RanBP5 were performed in the same buffer (50 mM Tris-HCl pH 8.0, 150 mM NaCl, 5 mM β-mercaptoethanol) using a S200increase (10/300 GL, GE Healthcare) column. UV

absorbance at 280 mm is plotted against the elution volume. MALLS-RI molecular mass estimates are plotted below the curve.

Supplementary Figure 7

Rg determination by Guinier extrapolation. Guinier plots were calculated on low q regions and are linear for (**a**) PA-PB1(1-686), (**b**) RanBP5 and (**c**) PA-PB1(1-686)-RanBP5.

Supplementary Figure 8

MONSA multi component fitting. MONSA builds a triple consensus *ab initio* model by simultaneously fitting the LnI(q) curve of (**1**)PA-PB1(1-686), (**2**)RanBP5 and (**3**)PA-PB1(1-686)-RanBP5.

Supplementary Figure 9

The 5'-vRNAp does not dissociate or bind to the PA-PB1(1-686)-RanBP5 complex. Prior to injection on SEC-MALLS, the RdRp-RanBP5 complex was incubated with a 3 fold molar concentration of 5'-vRNAp for 1h at 20°C. SEC-MALLS chromatograms are shown for both RdRp-RanBP5 wihtout (**a**) and with (**b**) 5'-vRNAp. Both analyses were performed in the same buffer (50 mM Tris-HCl pH 8.0, 150 mM NaCl, 5 mM β-mercaptoethanol).

References

1 Larkin, M. A. *et al.* Clustal W and Clustal X version 2.0. *Bioinformatics* **23**, 2947-2948, doi:10.1093/bioinformatics/btm404 (2007).

2 Robert, X. & Gouet, P. Deciphering key features in protein structures with the new ENDscript server. *Nucleic Acids Res* **42**, W320-324, doi:10.1093/nar/gku316 (2014).

3 Dias, A. *et al.* The cap-snatching endonuclease of influenza virus polymerase resides in the PA subunit. *Nature* **458**, 914-918 (2009).

4 Crepin, T. *et al.* Mutational and metal binding analysis of the endonuclease domain of the influenza virus polymerase PA subunit. *J Virol* **84**, 9096-9104 (2010).

Supplementary Figure 1

a Construct 12: PA-PB1-PB2

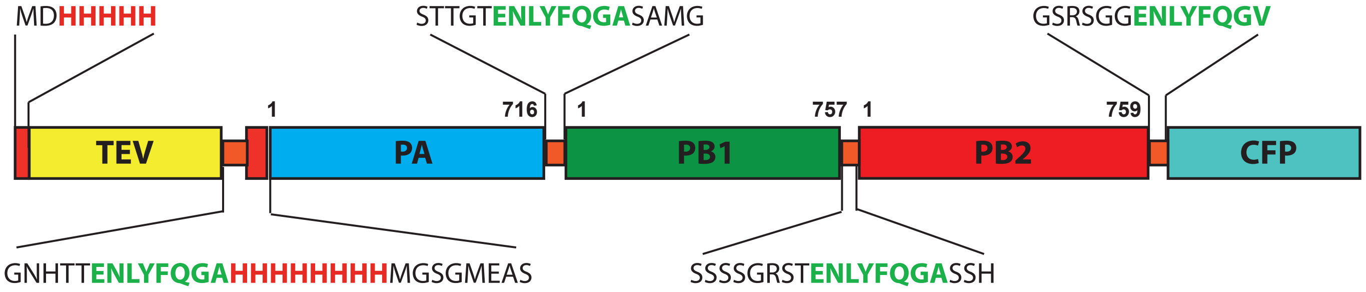

Protein	accession number
TEV	NP_734212.1
PA/H3N2	H9XIJ2
PA/H5N1	Q5EP34
PB1/H3N2	P31341
PB1/H5N1	Q5EP37
PB2/H3N2	H9XIJ5
PB2/H5N1	Q6DNN3
CFP	A0A059PIU2

b Construct 15: PA-PB1-PB2(1-116)

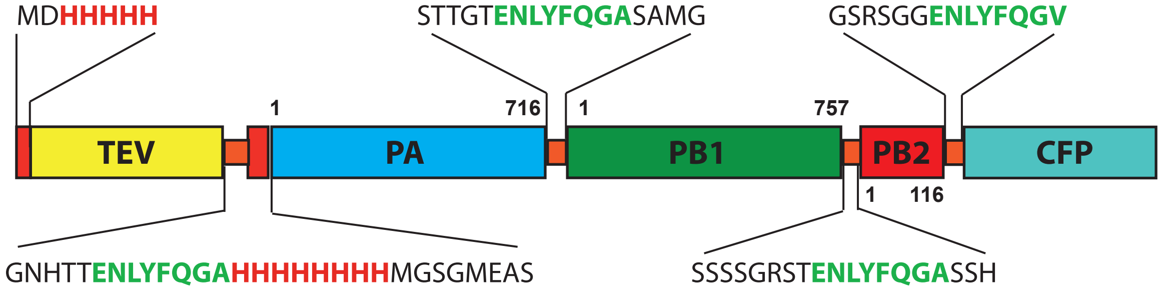

TEV	NP_734212.1
PA/H3N2	H9XIJ2
PA/H5N1	Q5EP34
PB1/H3N2	P31341
PB1/H5N1	Q5EP37
PB2/H3N2	H9XIJ5
PB2/H5N1	Q6DNN3
CFP	A0A059PIU2

c Construct 21: PA-PB1(1-686)-RanBP5

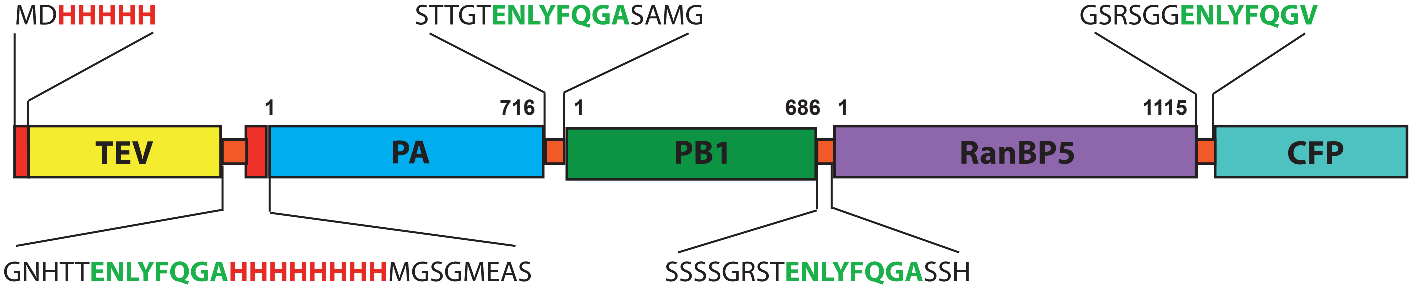

TEV	NP_734212.1
PA/H3N2	H9XIJ2
PB1/H3N2	P31341
RanBP5	CAA70103_1
CFP	A0A059PIU2

Supplementary Figure 2

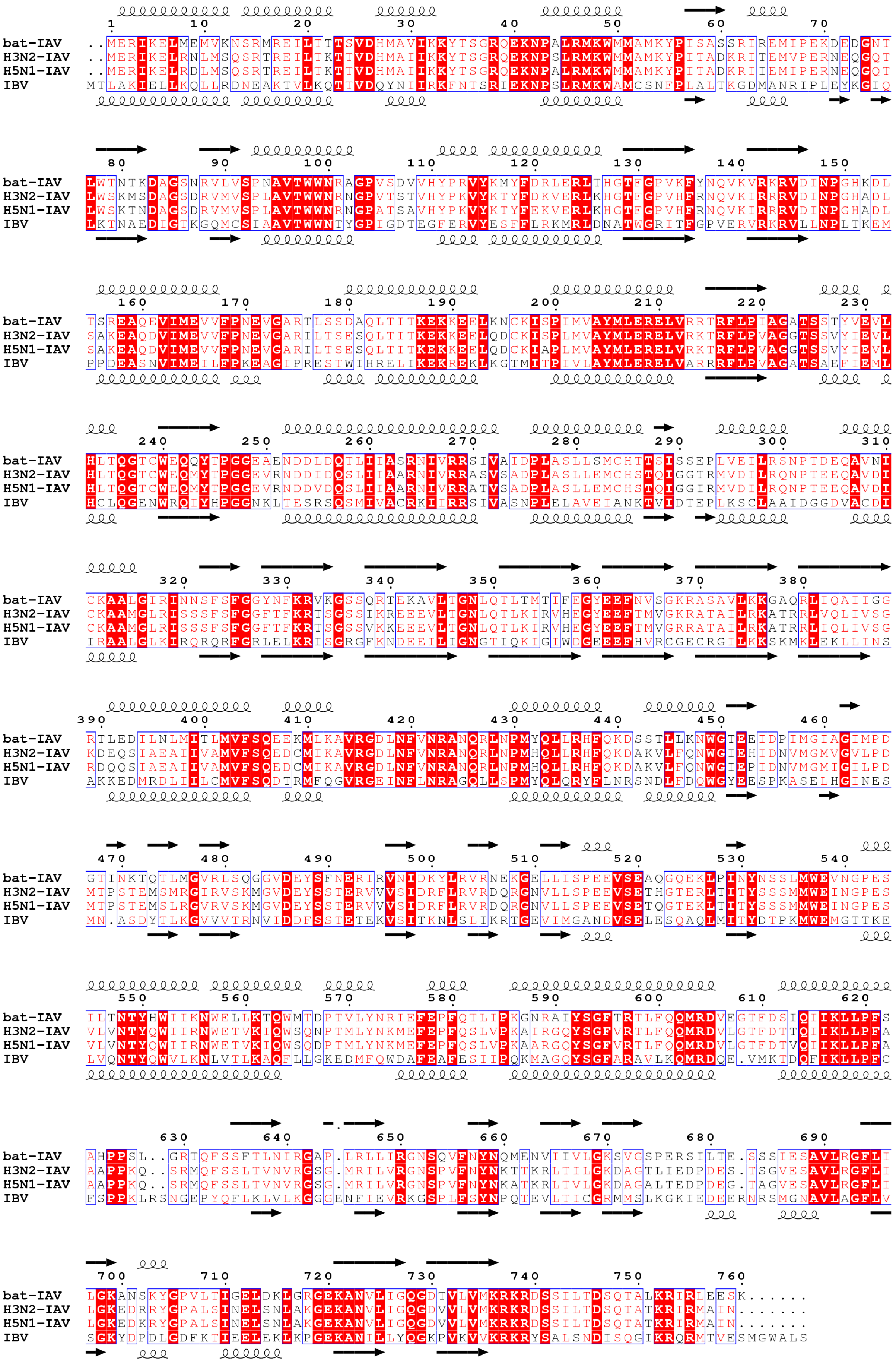

Supplementary Figure 3

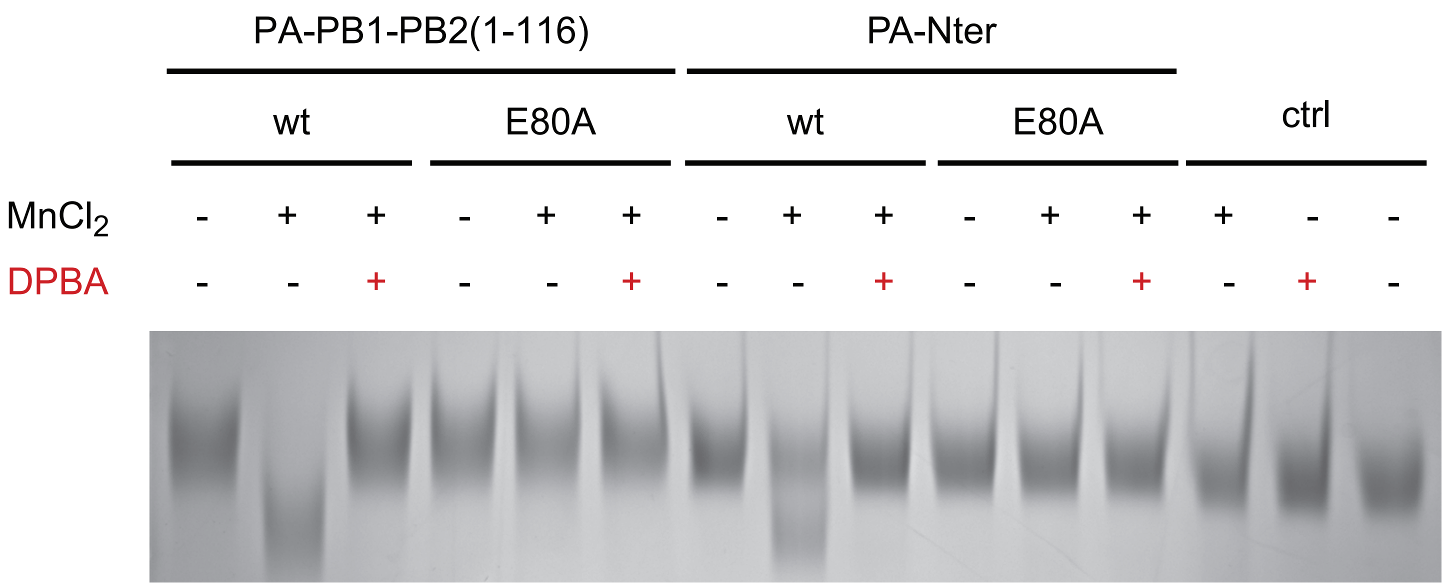

Supplementary Figure 4

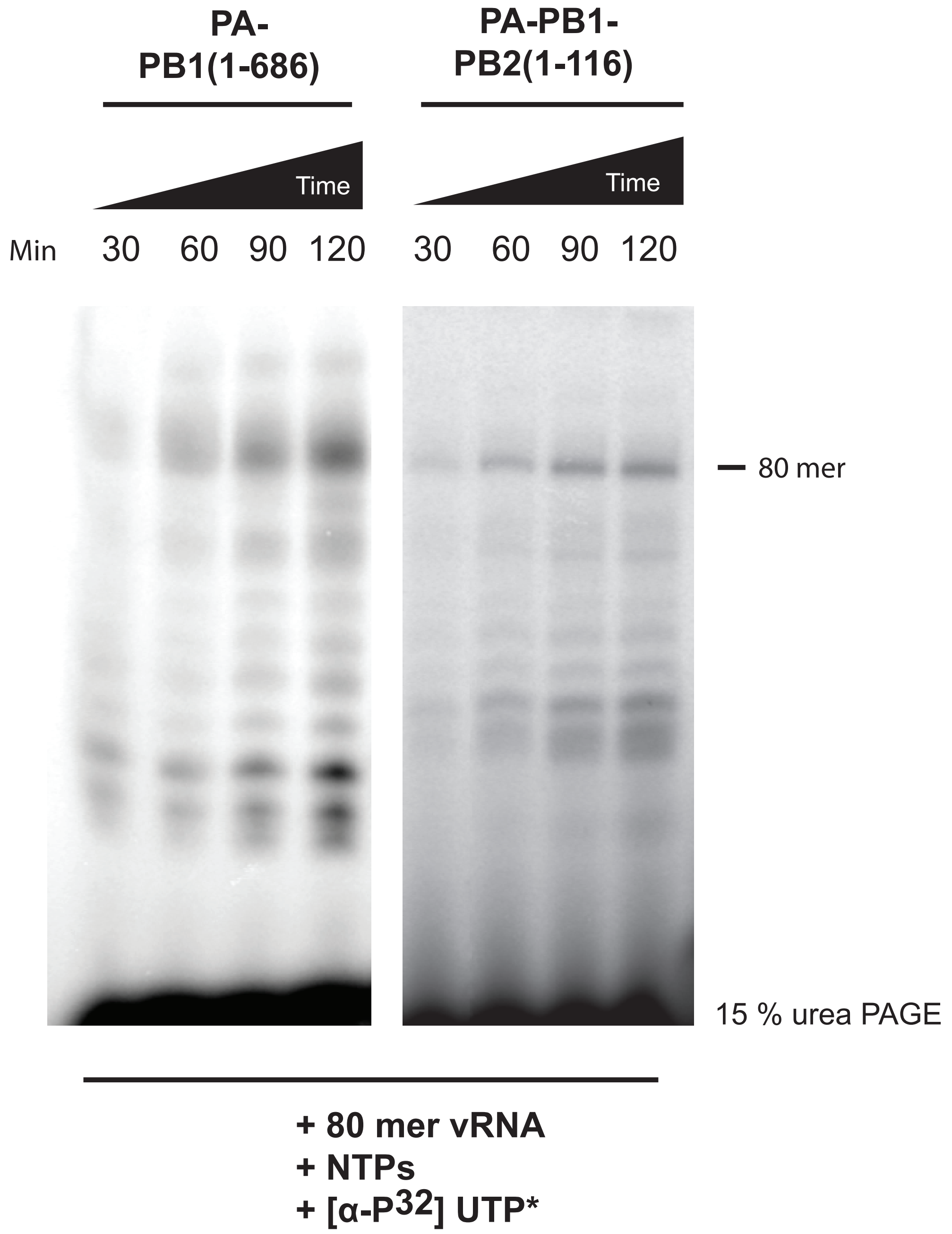

Supplementary Figure 5

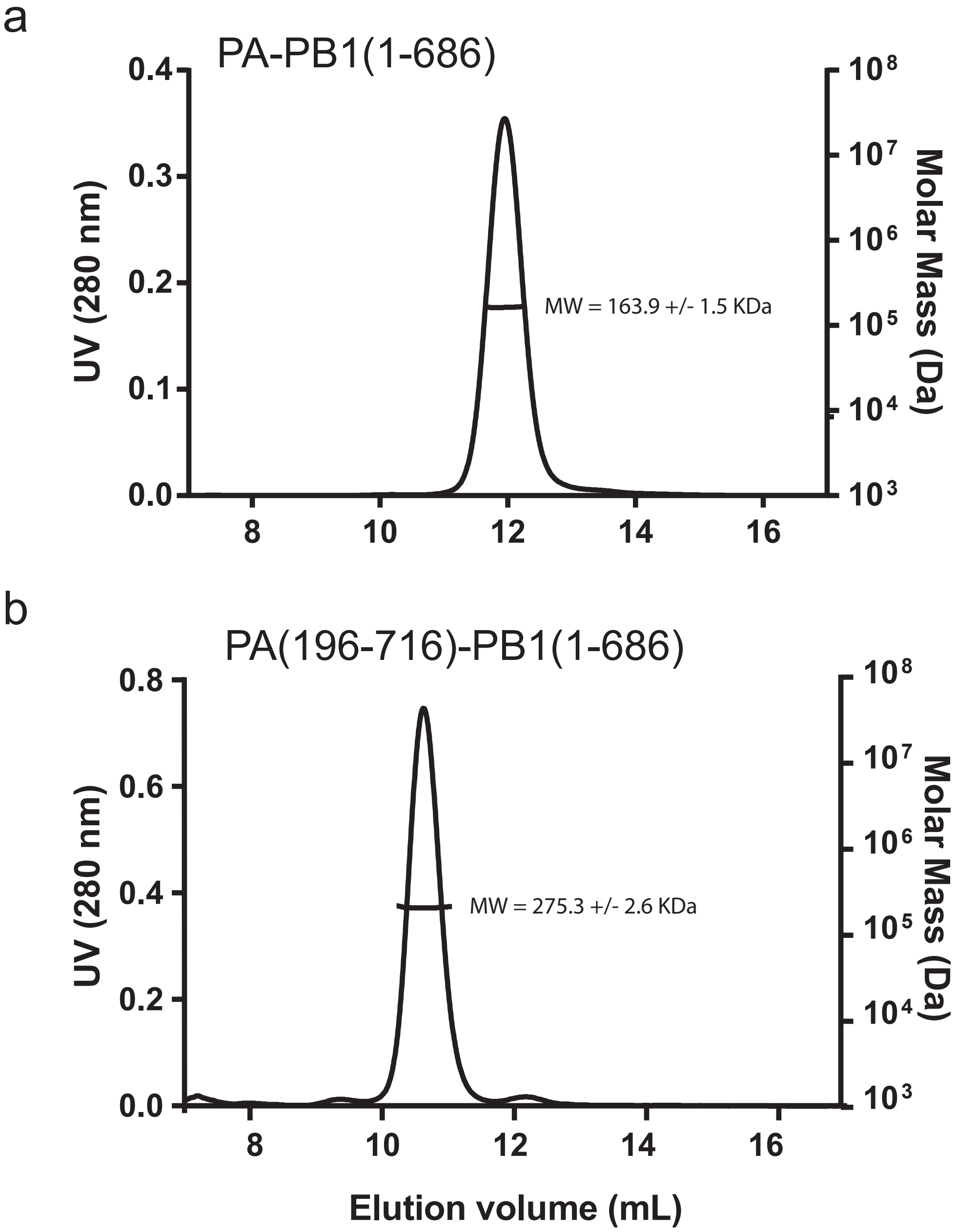

Supplementary Figure 6

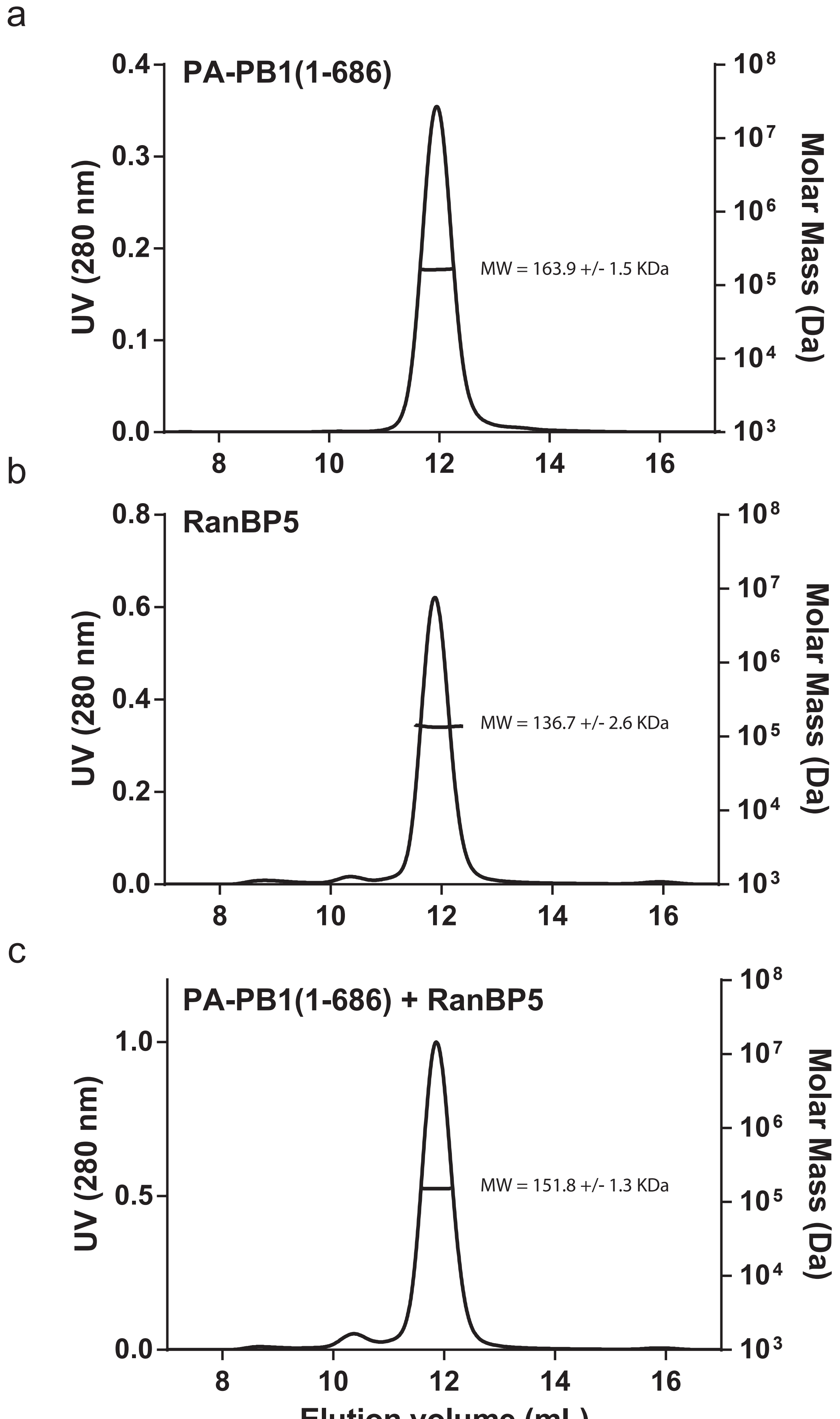

Supplementary figure 7

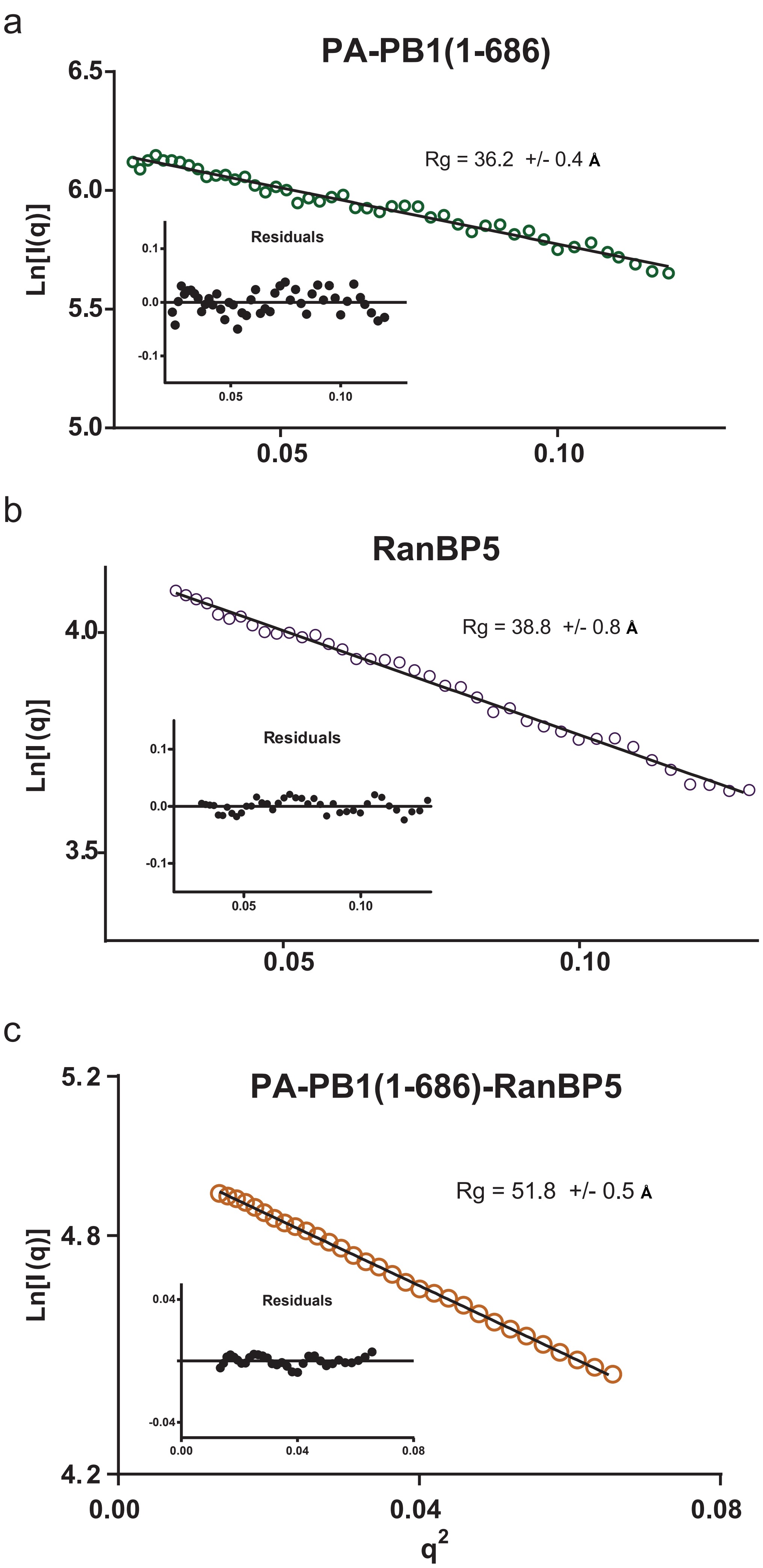

Supplementary figure 8

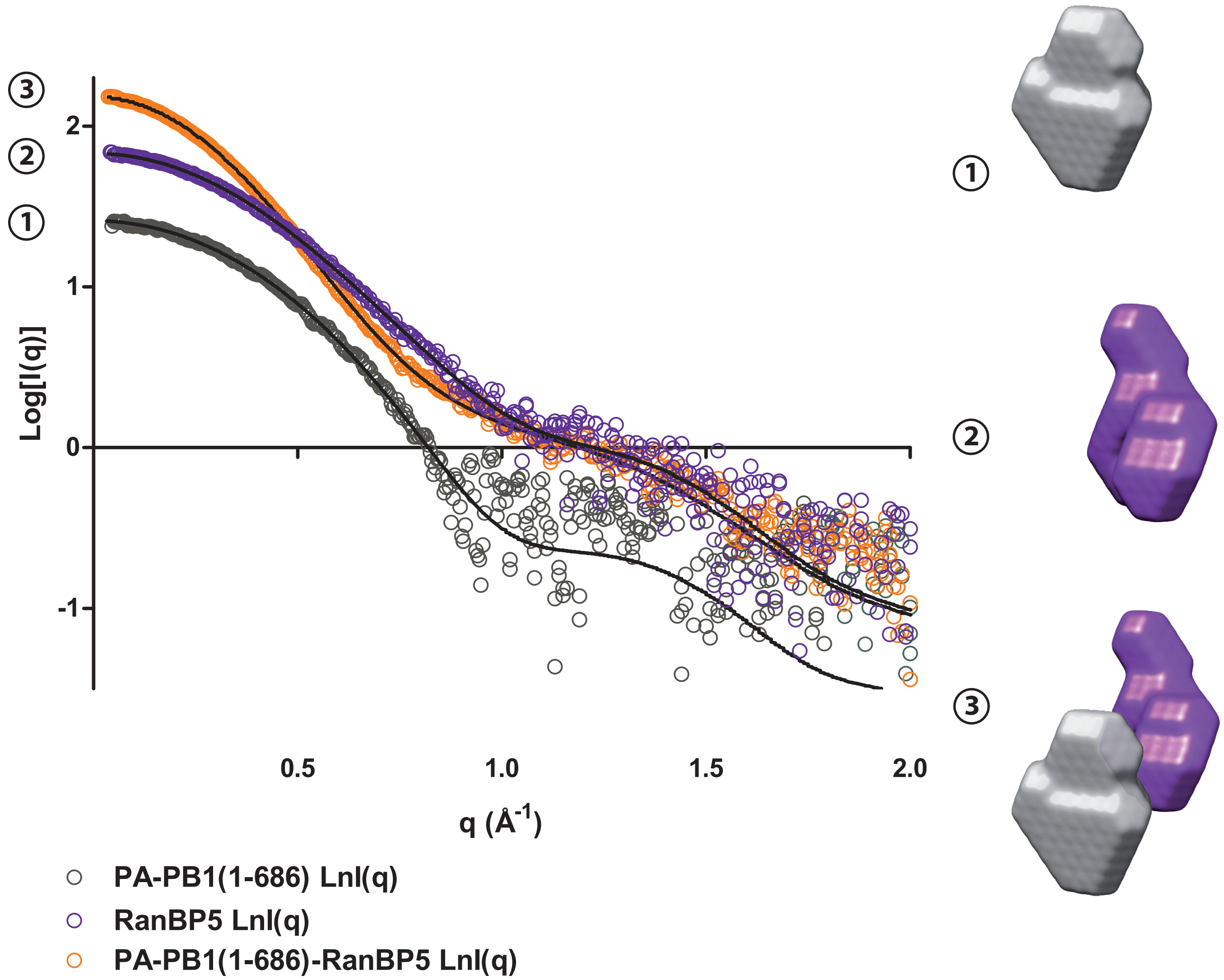

Supplementary Figure 9

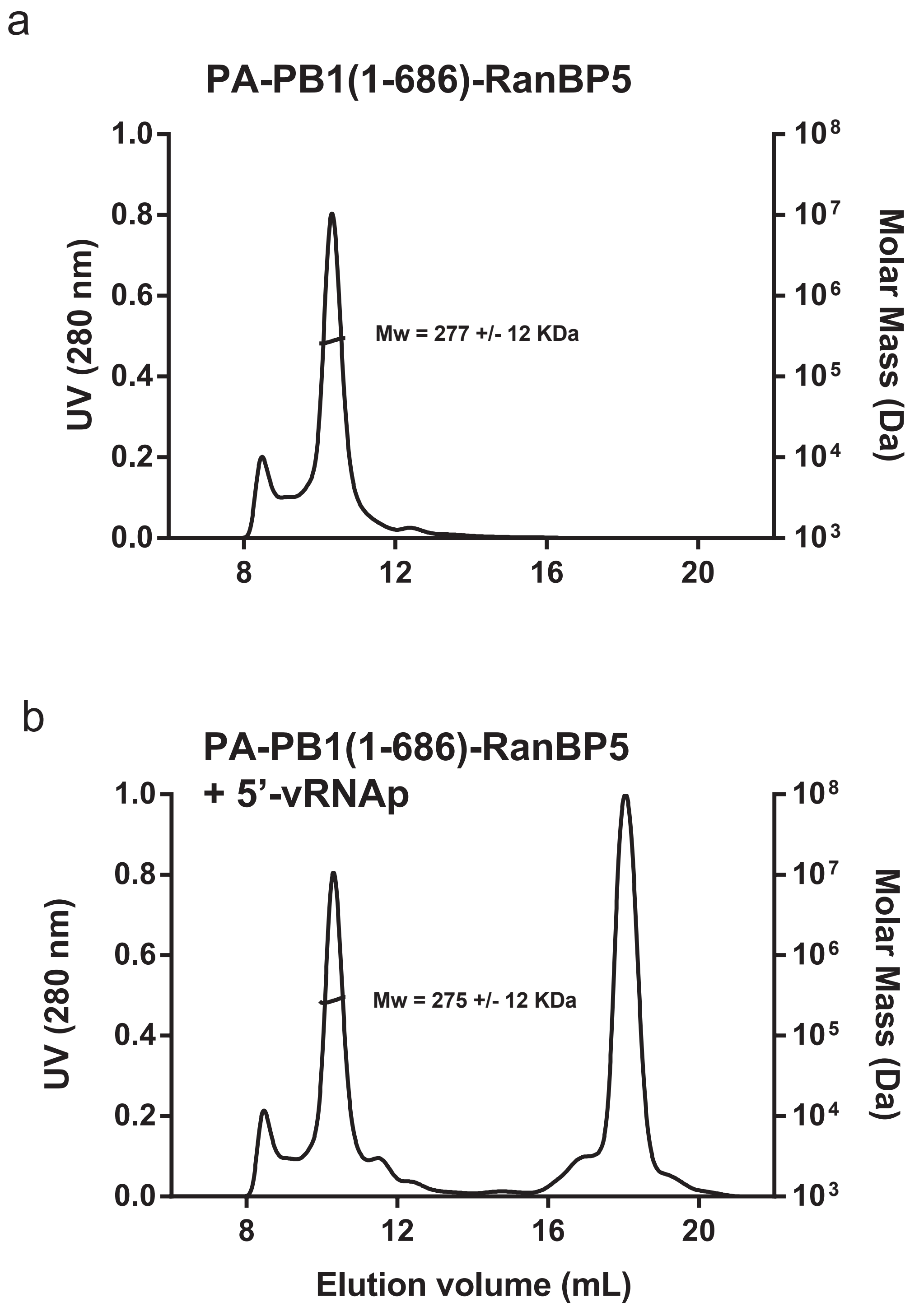

Supplement: Supplementary Information [file srep24727-s1.pdf]
